# Study on Cecropin B2 Production via Construct Bearing Intein Oligopeptide Cleavage Variants

**DOI:** 10.3390/molecules25041005

**Published:** 2020-02-24

**Authors:** Yi-Ting Fang, Si-Yu Li, Nien-Jen Hu, Jie Yang, Jyung-Hurng Liu, Yung-Chuan Liu

**Affiliations:** 1 Department of Chemical Engineering, National Chung Hsing University, Taichung 40227, Taiwan; tanya19931126@gmail.com (Y.-T.F.); syli@dragon.nchu.edu.tw (S.-Y.L.); 2 Innovation and Development Center of Sustainable Agriculture, NCHU, Taichung 40227, Taiwan; 3 Graduate Institute of Biochemistry, National Chung Hsing University, Taichung 40227, Taiwan; njhu@nchu.edu.tw (N.-J.H.); jieyang1110@gmail.com (J.Y.); 4 Institute of Genomics and Bioinformatics, NCHU, Taichung 40227, Taiwan; 5 PhD program in Medical Biotechnology, NCHU, Taichung 40227, Taiwan; 6 Department of Life Sciences, NCHU, Taichung 40227, Taiwan

**Keywords:** chitin-binding domain, intein, oligopeptide cleavage variants, purification, molecular dynamics simulation, first-order rate equation

## Abstract

In this study, genetic engineering was applied to the overexpression of the antimicrobial peptide (AMP) cecropin B2 (cecB2). pTWIN1 vector with a chitin-binding domain (CBD) and an auto-cleavage Ssp DnaB intein (INT) was coupled to the cecB2 to form a fusion protein construct and expressed via *Escherichia coli* ER2566. The cecB2 was obtained via the INT cleavage reaction, which was highly related to its adjacent amino acids. Three oligopeptide cleavage variants (OCVs), i.e., GRA, CRA, and SRA, were used as the inserts located at the C-terminus of the INT to facilitate the cleavage reaction. SRA showed the most efficient performance in accelerating the INT self-cleavage reaction. In addition, in order to treat the INT as a biocatalyst, a first-order rate equation was applied to fit the INT cleavage reaction. A possible inference was proposed for the INT cleavage promotion with varied OCVs using a molecular dynamics (MD) simulation. The production and purification via the CBD-INT-SRA-cecB2 fusion protein resulted in a cecB2 yield of 58.7 mg/L with antimicrobial activity.

## 1. Introduction

In recent decades, public misunderstanding about the use of antibiotics, the lack of strict distribution standards between clinics, and the addition of antibiotics to animal feed have led to an abuse of antibiotics, resulting in a rapid increase in antibiotic bacterial resistance. Researchers are, therefore, committed to developing new forms of antibiotics to replace the current pharmaceutical options. Existing data show that antibacterial peptides are the most promising candidates. Antimicrobial peptides (AMP) possessing antibiotic properties are widely distributed in nature, including in animals [1], insects [2], fish [3], crustaceans, and plants [4]. In general, AMPs can be obtained in three ways— extraction from organisms, chemical synthesis, or via genetic engineering. Although the first two methods result in AMPs with higher purity, the cost is prohibitively high and unsuitable for mass production. Therefore, a genetically engineered approach shows comparatively greater promise as a means to express large amounts of AMPs via microbial fermentation.

Cecropin is a non-specific AMP mostly derived from insects, arthropods, and invertebrates [5]. It is composed of 35–39 amino acids and has an α-helical structure. In the cecropins family, cecropin B (cecB2) was been found to be effective against not only specific Gram-positive and Gram-negative bacteria, but also some cancers in mammalian and non-mammalian cells [6]. The antimicrobial mechanism is due to the interaction between cecB2′s positive charge and the bacterial membrane’s negative charge [7]. In addition, the potential and ion concentration gradients between the inside and outside of the cell membrane causes the intracellular fluid to leak, eventually leading to cell necrosis [8,9].

Some studies reported on the use of microbes as hosts for the production of AMPs [10,11,12,13,14,15,16]; the major concern with this approach is that AMPs could prove toxic to their hosts. While the chitin-binding domain (CBD)-intein (INT) (CBD-INT) domain of pTWIN1 vector (NEB, USA) was helpful in cecB2 production [17], a key issue for this process was how to accelerate the pH-inducible INT self-cleavage reaction. INT self-cleavage is highly dependent on the amino acids adjacent to the INT (the +1 position of the target protein) [18]. According to the NEB manual, the sequences N↓CRA or N↓GRA (where arrows denote cleavage sites) are recommended for use with INT. Different oligopeptide cleavage variants (OCVs) have been suggested and utilized in recombinant protein production. Yan et al. proposed the use of the N↓C sequence to express human interferon-α4 (N↓CDI) [19]. Xu et al. suggested that the N↓G (N↓GRA) arrangement be used in the expression of the Buthus martensii Karsch insect toxin [20]. Zhang et al. produced the microbial polyhydroxyalkanote synthesis repression protein PhaR via the N↓GRA sequence [21], and Yan et al. expressed human interleukin-10 via the N↓GPG arrangement [22]. In addition, the N↓S sequence has also been observed in the literature. Sun et al. produced human brain natriuretic peptides via N↓SPK [23]. Esipov et al. expressed human epidermal growth factor via N↓SDS [24], and Setrerrahmane et al. synthesized lunasin via N↓SKW [25]. Many peptides and proteins have been produced via INT self-cleavage reactions with various OCVs; however, none of the studies has compared the effect of various OCVs for the same target protein production.

In our previous study, the production of cecB2 peptide was performed with the construction of a fusion protein with CBD-INT [17]. We found that the INT cleavage reaction was the rate-limiting step of the whole process. To accelerate the INT self-cleavage, the insertion of a peptide segment (OCV) in the INT C terminus was proposed. However, there was no guide to decide which OCV was the best. Therefore, in this study, we tried to study the INT self-cleavage effect with the aid of OCVs. Due to the convenience of the test, we just used the commonly used LB (Luria-Bertani) medium to cultivate the strain. Three OCVs, i.e., GRA (Gly-Arg-Ala), CRA (Cys-Arg-Ala), and SRA (Ser-Arg-Ala), which were frequently used in the prior studies, were adopted. The pH-dependent cleavage efficiency was evaluated and compared. The effects of pH and temperature on INT self-cleavage were also explored. In addition, a first-order rate equation was applied to predict the INT self-cleavage kinetic behavior. To understand the promotion effect, an in silico structural analysis of the OCVs was carried out using a molecular dynamics (MD) simulation. Finally, the use of OCVs to accelerate INT cleavage in cecB2 production was illustrated.

## 2. Results

### 2.1. Gene Construction

**Figure 1:**
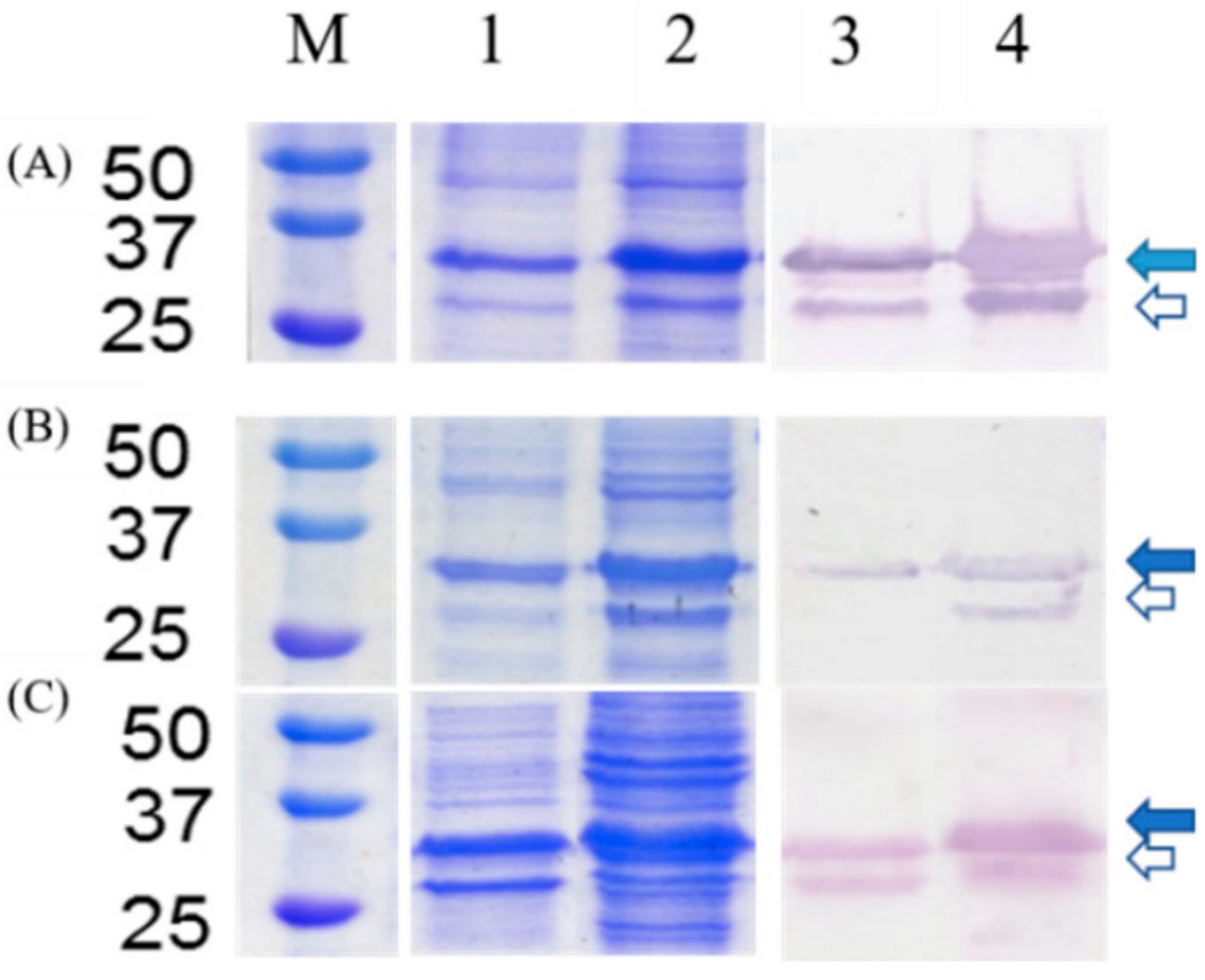
SDS-PAGE and Western blot analysis of pTWIN1-chitin-binding domain (CBD)-intein (INT)-oligopeptide cleavage variants (OCV)-cecropin B2 (CecB2): Lane M: marker (kDa), Lane 1–2: pellet and supernatant of the SDS-PAGE, Lane 3–4: pellet and supernatant of Western blot, where the OCV is (**A**) GRA, (**B**) CRA, and (**C**) SRA. Blue arrows show the fusion protein and white arrows show the cleaved part location.

To construct the fusion proteins, a T vector carrying the cecB2 gene was used as a template and various primers were designed (Table 1) to amplify the cecB2 gene via PCR. The PCR products were further coupled to vector pTWIN1 with the aid of restriction sites, as shown in Figure 1. According to NEB’s product manual, the use of oligopeptides G-R-A or C-R-A at the N-terminus of the target protein could facilitate INT C-terminal splicing-region cleavage. In addition, Ser and Cys with the respective hydroxyl and thiol side chains could serve as nucleophilic amino acids to promote INT cleavage [19,23,24]. Therefore, three OCVs, i.e., GRA, CRA, and SRA, were applied in this work to explore their effect on facilitating INT self-cleavage. Figure 1 shows the schematic map for the constructs of the recombinant vectors pTWIN1-CBD-INT-OCV-cecB2. *Escherichia coli* ER2566 was selected as the host due to its lack of outer membrane proteases [5]. The three constructs were transferred to *E. coli* ER2566 competent cells via heat shock.

### 2.2. Fusion Protein Expression

To test the recombinant constructs, the recombinant strains with various pTWIN1-CBD-INT-OCV-cecB2 were subjected to protein expression following the procedure outlined in Section 2.3. The cells were cultivated in the LB medium at 37 °C and 200 rpm until a cell density of OD = 0.6 and then induced with 0.4 mM IPTG (isopropyl β- d-1-thiogalactopyranoside), followed by incubation at 15 °C and 200 rpm for 16 h. The harvested cells were subjected to sonication and centrifugation. The supernatants and pellets were collected for comparison of protein expression via SDS-PAGE and CBD Western blot analysis. For the three constructs, fusion proteins with a molecular size of 29 kDa (8 kDa for CBD, 17 kDa for INT, OCV << 1 kDa, and 4 kDa for cecB2) were clearly observed in PAGE and CBD Western blot analysis, as shown in Figure 1. It was found that a large amount of soluble protein could be harvested in the supernatant. However, there were still some levels of the inclusion body in the pellet of the cell lysis broth even at the reduced induction temperature. Meanwhile, the expression of CBD-INT domain was also observed as the band with a molecular size of 25 kDa. It is not possible that the self-cleavage reaction occurred in the fusion protein expression stage due to the toxicity of the cecB2 to the cells. This effect was further confirmed via checking the PAGE gel, and no band appeared at the lower molecular weight down to 4 kDa. It is assumed that the incomplete fusion protein expression or the post-harvest cell lysis process might have been attributed to the CBD-INT segment in the SDS-PAGE.

### 2.3. Determining Cleavage Timescale for Various OCV

**Figure 2:**
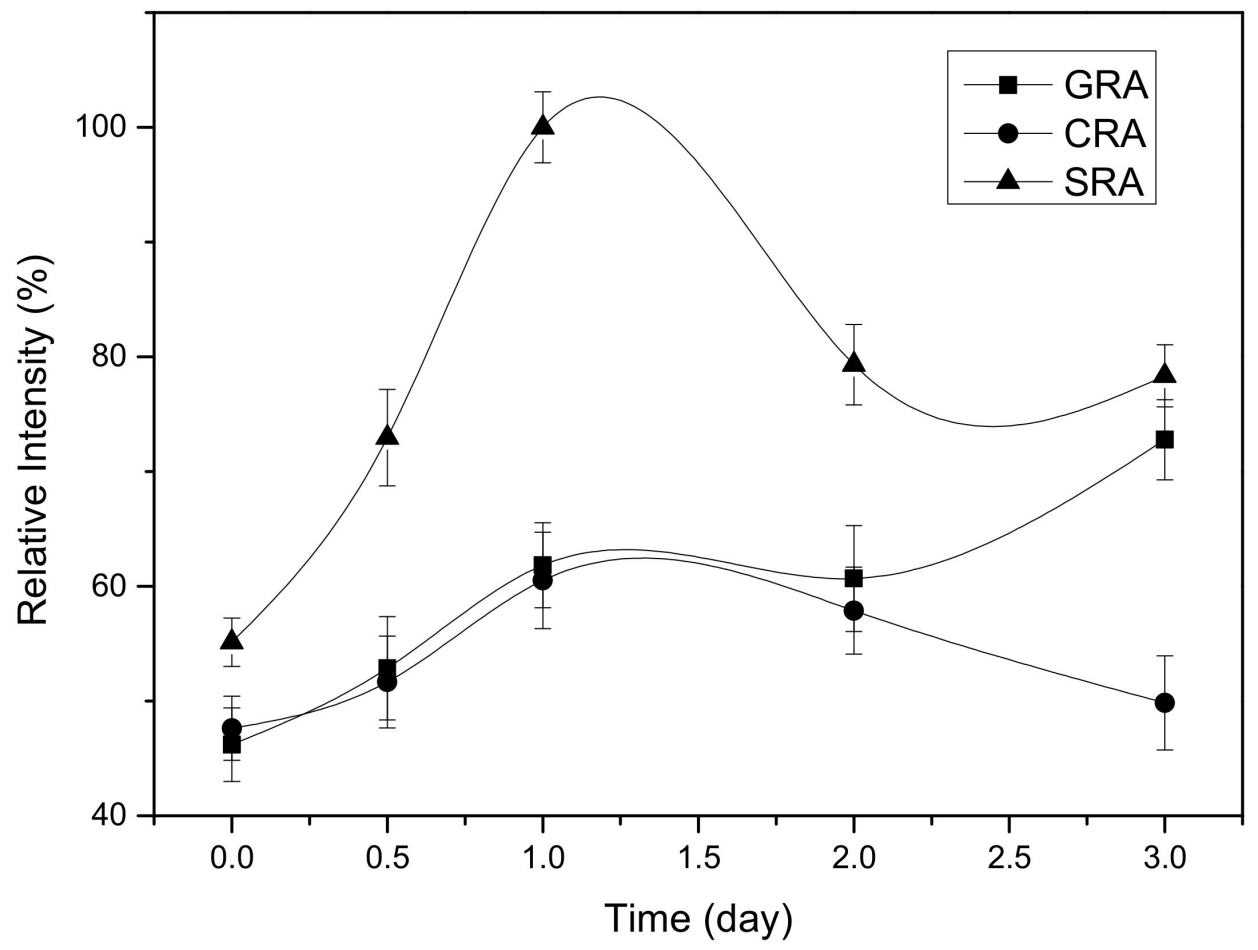
Time profiles of the intein cleavage reactions for three OCVs, where the relative color intensity of the cleaved part was used to represent the protein concentration.

For the INT cleavage test, the harvest supernatant was subjected to filtration to remove the proteins with a molecular weight of less than 10 kDa, as depicted in Section 4.4. Due to the removal of the small molecular peptides (including the product cecB2) prior to the test, the cleavage reaction might not have been subjected to product inhibition. In addition, the high-level expression of the fusion protein was expected to minimize the influence of other impure proteins on the reaction. To practice the cleavage test, the upper retentate was mixed with the cleavage buffer (20 mM Tris HCl, 50 mM NaCl) as per NEB’s instructions. After the INT cleavage reaction, the cleaved part of the fusion protein in SDS-PAGE was scanned and the color intensity was measured to quantify the cleavage level. It also indicated that the most suitable pH for INT cleavage at the C-terminus was pH 6.0–7.5 [18]; a pH value higher than 8.0 or lower than 5.5 would have suppressed the cleavage reaction. As the optimal conditions for the cleavage were still not performed, the conventional practice for INT cleavage was applied for the comparison of its cleavage with various OCVs. In this test, the pH was adjusted to pH 6.5 and the temperature was set at 25 °C. Various OCVs were first time tested to facilitate the intein cleavage reaction. Each cleavage reaction was performed for 3 d in triplicate. The results of the color intensity of the cleaved parts were measured and normalized as shown in Figure 2. It was found that the intein with SRA gave the most efficient result in the cleavage reaction. In addition, the cleavage reaction could be done within 1 d. As for the GRA and CRA, the reaction rates were significantly lower than that of SRA, and in addition, a longer process might have yielded a negative result for SRA and CRA.

### 2.4. Kinetic Study of INT Cleavage Reaction

**Figure 3:**
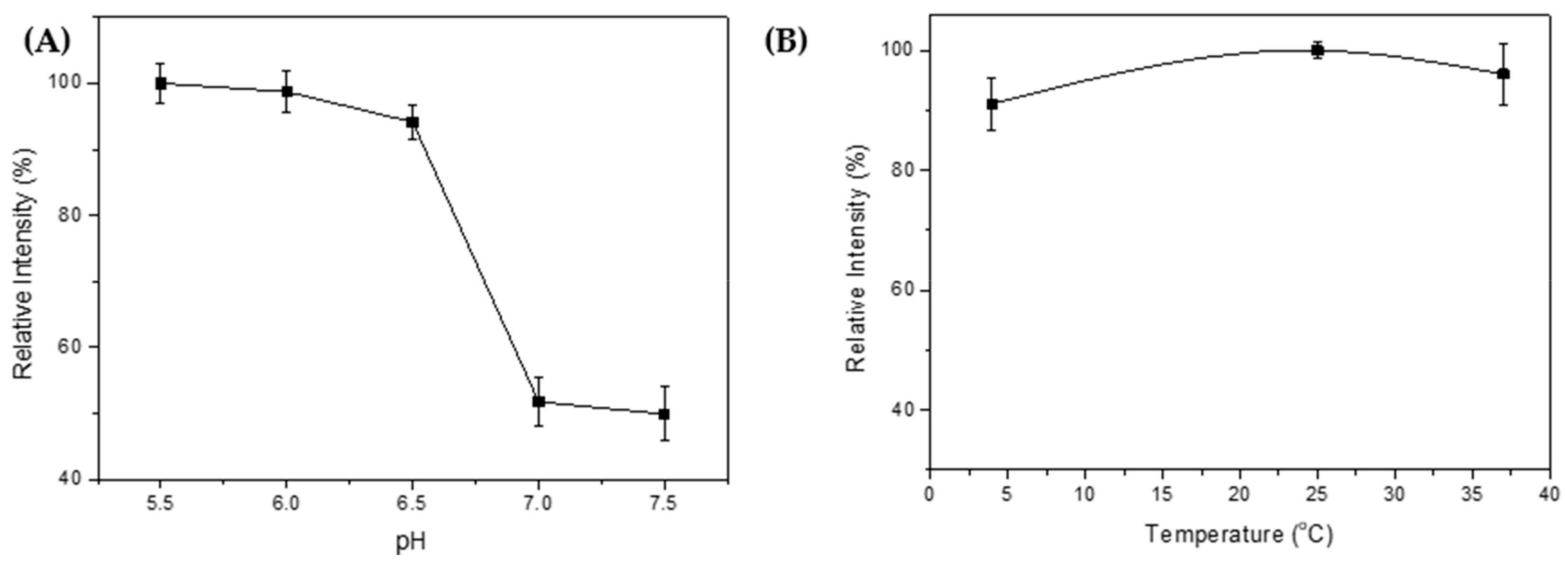
(**A**) Effect of pH on intein self-cleavage, (**B**) Effect of temperature on intein self-cleavage, where the relative intensity was calculated by dividing the color intensity by the highest color intensity.

In this study, an attempt was made to find out whether INT could be treated as a catalyst for the self-cleavage reaction; in this way, the kinetics of INT cleavage reactions were tested. From the time courses of the cleavage reactions for various OCVs shown in Figure 2, it was seen that all the fusion proteins showed a positive cleavage relationship from 0–24 h. Therefore, it is quite feasible to express the INT cleavage reaction with a first-order rate equation. According to Equation (2), the semi-log plot of color intensity with time was drawn and a linear regression was performed. The slope of the fitting equation was the rate constant. The results are displayed in Table 2. The rate constants followed the order of SRA > GRA > CRA, which quantitatively agreed with the observation of the cleavage reaction in Figure 3. This means that treating intein as a biocatalyst is quite feasible. It was also found that a longer cleavage time, from 24 to 72 h, resulted in an obvious downward trend in the cleavage, perhaps due to the protein degradation that occurs after an extended period of time. In order to avoid any loss in proteins, the cleavage time was set to 1 d.

### 2.5. Intein Cleavage Conditions

In order to determine the best conditions, the fusion protein CBD-INT-SRA-cecB2 was selected and subjected to various cleavage conditions (i.e., pH and temperature). As per NEB’s instructions, a cleavage buffer (20 mM Tris HCl, 50 mM NaCl) was used. It also indicated that the most suitable pH for cleavage at the C-terminus was pH 6.0–7.5; a pH higher than 8.0 or lower than 5.5 would have suppressed the cleavage reaction. In the literature, the optimal condition for cleavage was reported to be pH 7.0 [18]. The mixtures were allowed to stand at 4 °C for 1 d before the samples were subjected to SDS-PAGE analysis. The color intensity of each cleaved fragment was analyzed via ImageJ and is shown in Figure 3A. According to the results, the cleavage reaction was clearly suppressed at a pH of around 7.0–7.5. In contrast, the INT cleavage reaction functioned well at pH 5.5–6.5, and a little lower pH seemed better for the cleavage reaction. This result differs from the reports that a pH range of 6.0–7.0 is the best for cleavage [18]. In this study, the cleavage could be significantly inhibited when the pH was higher than 6.5.

To test the effect of temperature on INT cleavage, various temperatures were applied (4, 25, and 37 °C). The cleavage reaction was set at pH 6.0 for 1 d. The results are shown in Figure 3B. It was found that there were no significant differences in reaction at temperatures ranging from 4–37 °C. For a convenient operation, the ambient temperature of 25 °C was chosen for the following cleavage reaction.

### 2.6. In-Column Purification for CecB2 Production and Its Activity

**Figure 4:**
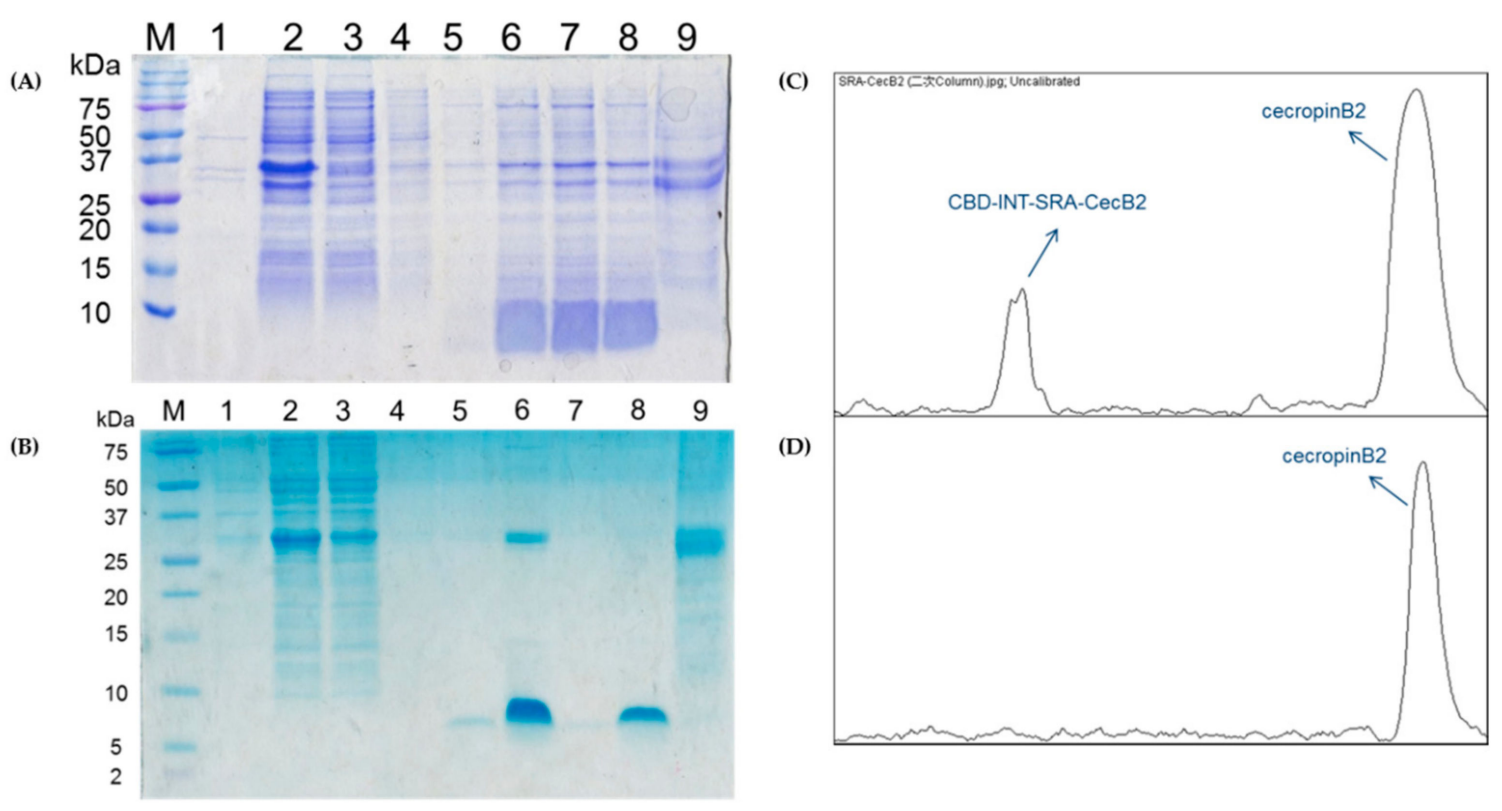
Purification of pTWIN1-CBD-INT- SRA-CecB2 in (**A**) SDS-PAGE—Lane M: marker (kDa), Lane 1: pellet, Lane 2: supernatant, Lane 3: flow through, Lane 4: cleavage buffer washing, Lane 5: elution 1, Lane 6: 10x elution 1, Lane 7: 10x elution 2, Lane 8: 10x elution 3, and Lane 9: striping; (**B**) Tricine-SDS-PAGE—Lane M: marker (kDa), Lane 1: pellet, Lane 2: supernatant, Lane 3: flow through, Lane 4: washing, Lane 5: elution, Lane 6: 20x elution, Lane 7: elution via reloading, Lane 8: 20x of Lane 7, and Lane 9: striping; (**C**) purity analysis of Lane 6; and (**D**) Lane 8 via ImageJ scanning.

To test the in-column purification of the fusion proteins after the *E. coli* cultivation, the obtained supernatant with the fusion protein CBD-INT-SRA-cecB2 was subjected to a CBD affinity column, followed by washing, elution, and stripping procedures. The results of the SDS-PAGE are shown in Figure 4A. It was found that the fusion proteins could be adsorbed onto the chitin resin. During the washing process, some impure proteins were flushed away. In the cleavage reaction, the cleavage buffer with a pH of 6.0 was loaded and left to stand overnight at 25 °C. The eluted sample containing cecB2 was collected and analyzed. A smear band of below 10 kDa for the 10-fold concentrated elution samples was found. The smear effect was due to the use of 15% PAGE, which caused band diffusion for small molecule-weight peptides. To clearly detect cecB2, the same procedure was performed and the results were displayed in Tricine-SDS-PAGE, as shown in Figure 4B, where the cecB2 peptide exhibited a clear band, indicating that this approach is proper for cecB2 production. In addition, it was found in Figure 4B that the eluent in elution flow would contain not only cecB2 but also some of the fusion protein, as represented in Figure 4C (scanning via ImageJ). It was assumed that the low pH of the cleavage buffer (pH 6.0) might be attributed to the desorption of fusion protein. To remove the carrying fusion protein, the eluent was reloaded into the column. High purity cecB2 without any impure proteins was obtained in the elution stream (Figure 4D). While the reload process did remove the fusion protein, it also trapped some cecB2 in the column and caused a reduction of the cecB2 yield. To quantitatively determine the cecB2 concentration, the color intensity was measured via ImageJ and correlated to a BSA standard curve—a cecB2 yield of 58.7 mg/L was obtained.

In previous reports, the cecropin family was reported to exhibit various antimicrobial effects against Gram-negative strains, Gram-positive strains [26] and even cancer tumors [27]. To prove the antimicrobial activity of the cecB2 produced in this study, a serial dilution test was applied against three *E. coli* strains. The serial dilution number of the purified cecB2 (58.7 mg/L) was detected in triplicate and is shown in Table 3. It was found that the average serial dilution number was in the order of *E. coli* JM109 > *E. coli* BL21 > *E. coli* DH5α. Although the antibacterial activity might be a little different for the strains belonging to the same species, in this type of assay, minimum inhibition concentration (MIC) values differing by a factor of two are assumed to be identical. The calculated MIC values were in the range of 1.8–3.7 (mg/L), equivalent to 0.45–0.90 μM. Wang et al. reported that the synthesized cecropin B showed an MIC of 0.78–1.56 μM to various *E. coli* strains [28], which is quite consistent with that observed in this study.

## 3. Discussion

**Scheme 1:**
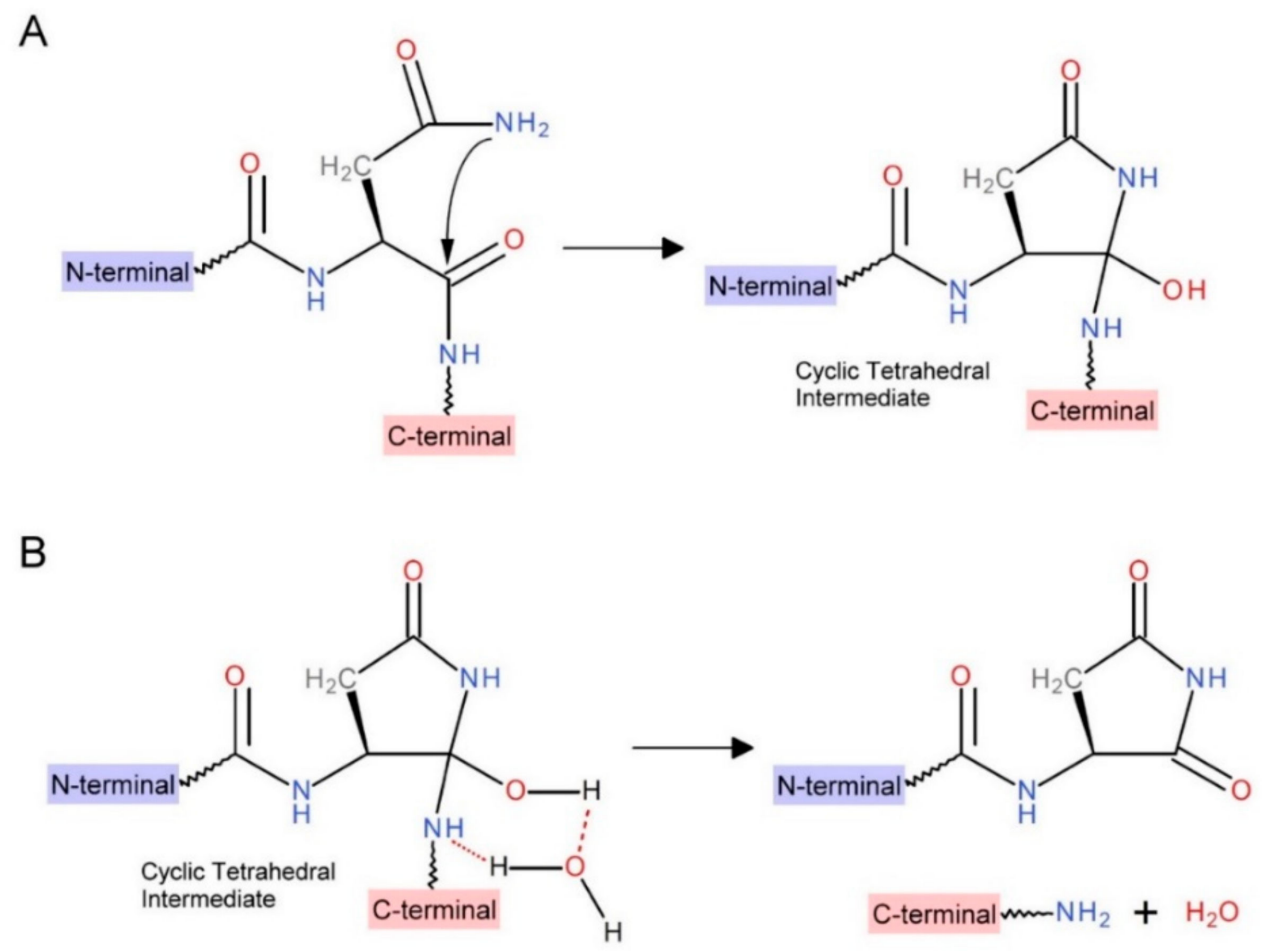
Proposed chemical mechanism of pH-dependent cleavage at Ssp DnaB intein C-terminus: (**A**) sidechain NH_2_ attack on Asn backbone carbonyl to form a cyclic tetrahedral intermediate; (**B**) water-assisted amine bond breaking at the intermediate.

In this study, it was found that the fusion protein with an OCV of SRA inserted into the INT C-terminus was shown to promote the self-cleavage reaction of the intein. Scheme 1 is prepared based on the quantum chemistry calculations proposed by Catak et al. According to this Scheme, the pH-dependent INT self-cleavage might occur via a two-step mechanism [29]. The first step is the cyclization of a conserved Asn residue at the C-terminus of the INT, forming a cyclic tetrahedral intermediate (Scheme 1A). The next step is the breaking the amine bond, due to the surrounding water (Scheme 1B). Catak et al. also proposed that the peptide bond cleavage at Asn residues is more likely to take place after it has deamidated into Asp, because Asn is known to be unstable at a pH value near 7 [29].

According to the scheme, the cleavage reaction may be affected by two factors—the structural stability of the cleavage sequence and the chemical environment near the peptide bond. A stable conformation of the cleavage sequence will help the cyclization of Asn via sidechain NH2 attack (Scheme 1A). In addition, a hydrophilic chemical environment near the peptide bond will attract water molecules to accelerate the peptide bond breakage (Scheme 1B).

**Figure 5:**
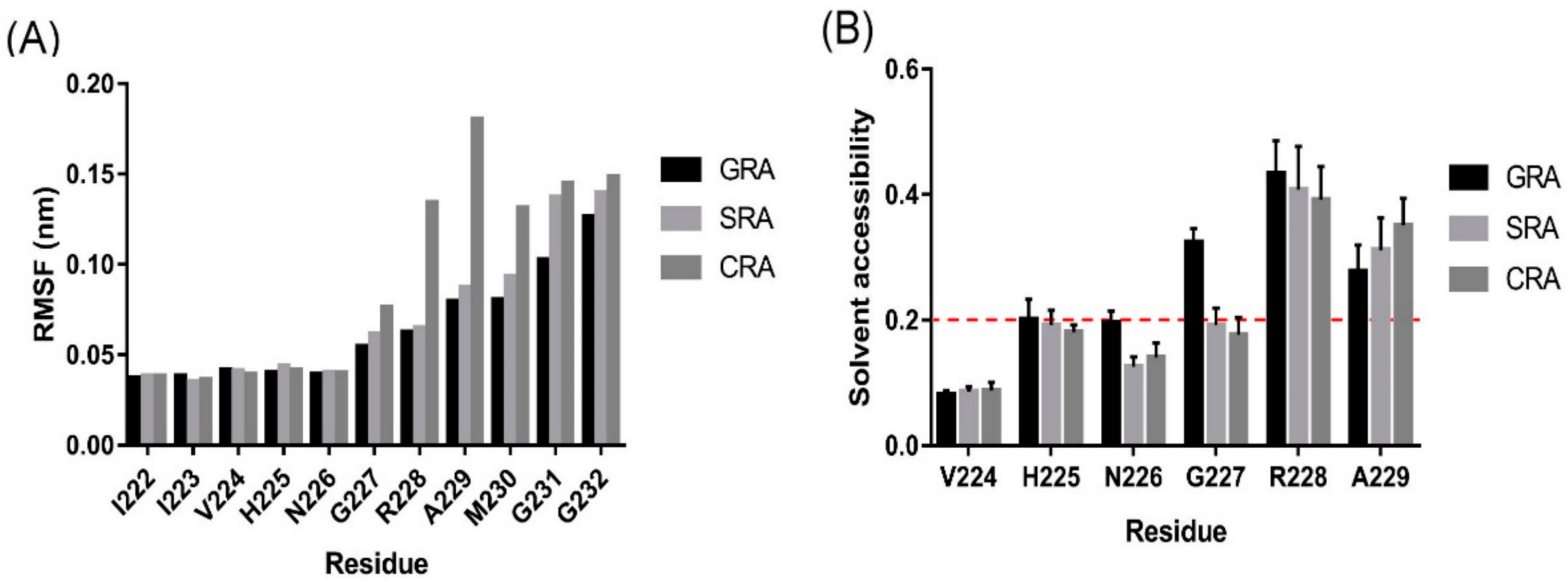
Comparative analysis of the dynamics of CBD-INT-OCV-cecB2 fusion protein variants: (**A**) the root-mean-square fluctuations (RMSF) of Cα atoms in the cleavage sequence calculated from the molecular dynamics (MD) simulations; (**B**) the solvent accessibility of residues in the cleavage sequence. The solvent accessibility was calculated from MD simulation snapshots. A cutoff of 20% (red dashed line) was used to define the two states—buried or exposed.

To explore the situation in the cleavage reaction, all-atom MD simulations of these OCVs were carried out. The structural stability of the cleavage sequence was evaluated via the root-mean-square fluctuations (RMSF) for Cα atoms from the 10-ns simulation trajectory files [30]. The RMSF values for the cleavage sequences of the three variants are shown in Figure 5A. SRA and CRA showed higher fluctuations in residues 227 to 232, while residues 228 to 230 in CRA exhibited a highly dynamic status compared to those in GRA and SRA. SRA and CRA also showed lower solvent accessibility in residues 226 and 227 (Figure 5B).

**Figure 6:**
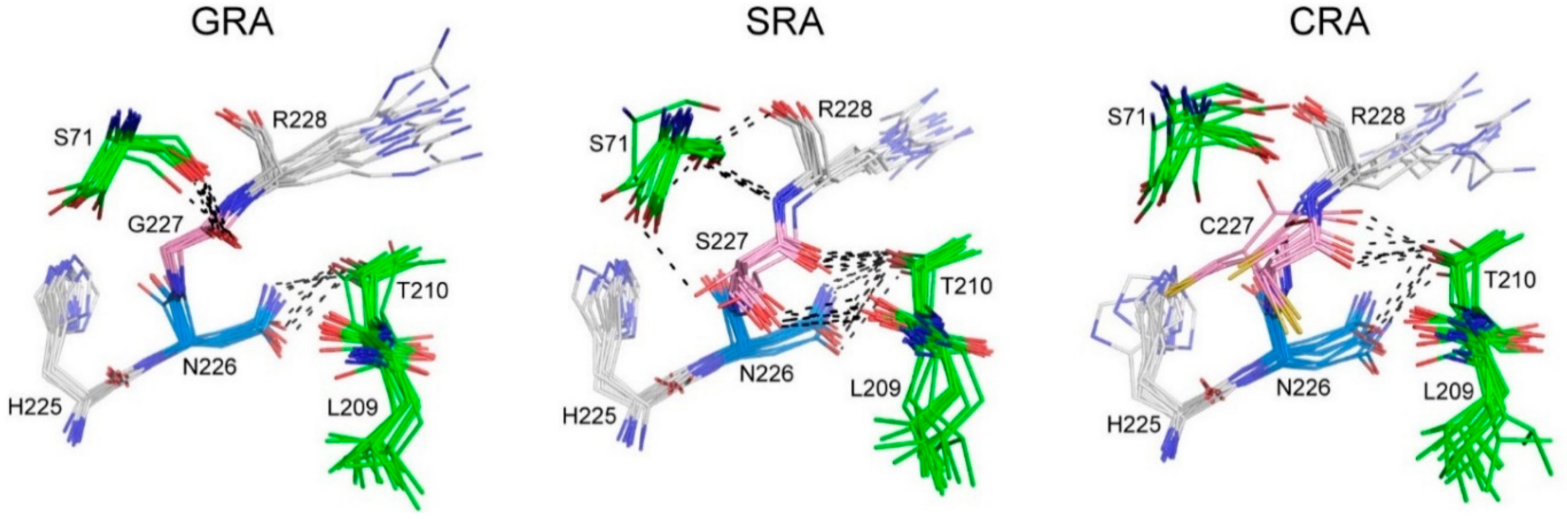
Conformational ensembles of the cleavage sites of CBD-INT-OCV-cecB2 fusion protein variants during 10 ns of MD simulation. For clarity, catalytic Asn (N226) and the +1 position residues (G227, S227, or C227) of OCV are colored in light blue and pink, respectively. The neighboring residues are in green. The potential hydrogen bonds are depicted as black dashed lines.

From the MD simulation snapshots (Figure 6), it was determined that G227 on GRA has little interaction with neighboring residues (S71, L209 and T210) due to its lack of sidechain. Conversely, S227 on SRA uses its polar sidechain to form hydrogen bonds with neighboring residues. C227 of CRA, on the other hand, makes non-specific van der Waals contact with neighboring residues, because the thiol (SH) group is a donor with weaker hydrogen bonds. Non-specific van der Waals contact increases the conformation diversity of the cleavage sequence; as a result, it decreases the structural stability of the cleavage sequence in CRA. Furthermore, the size of the sidechain and massive inter-residue interaction for residue 227 provide a closed environment for N226 in SRA and CRA, making it less accessible to the solvent (i.e., water). In summary, SRA has several structural features beneficial to the self-cleavage reaction. First, the stable conformation of the cleavage site (the region between residues 226 and 227) may facilitate the cyclization of Asn via the sidechain NH2 attack (Scheme 1A) and the water-assisted breaking of the amine bond (Scheme 1B). Second, a closed environment (less solvent accessibility) may help the cyclization of Asn as it provides steric effects to ensure the NH2 attack the Asn backbone carbonyl group. Third, the hydrophilic sidechain of S227 may attract surrounding water to accelerate the peptide bond breakage. Although the mechanism for the intein self-cleavage with OCVs could be practiced via other approaches, such as quantum mechanics/molecular mechanics calculations [31,32], the MD analysis in this study could still provide a simple and quick insight on the microenvironment molecular behavior for various OCV structures.

In this study, inteins with various OCVs were constructed and their effect on INT self-cleavage was tested for cecB2 production. When comparing various OCVs, SRA gave the best result for facilitating the INT cleavage reaction. Assuming INT as a biocatalyst, a first-order reaction rate equation was found to be a good tool for predicting the kinetics of the self-cleavage reaction. The in-column purification could be properly applied to the preparation of cecB2, expressing antimicrobial activity against various *E. coli* strains. In addition, some advantages for the construction of INT with SRA was highlighted via the MD simulation. In conclusion, the presence of the CBD-INT-SRA domain might not only accelerate the INT self-cleavage reaction, but also make product purification easier via the in-column cleavage reaction. The process developed in this study may provide a reference for other similar AMP production and for future large-scale applications.

## 4. Materials and Methods

### 4.1. Strains, Plasmids, and Cecropin B2

*E. coli* DH5α and *E. coli* ER2566 were used as host cells for gene transfer and recombinant protein expression, respectively. The plasmid pTWIN1 containing the CBD-INT gene sequence and the mouse monoclonal anti-CBD were obtained from NEB (New England Biolabs Inc., Hitchin, UK). The plasmid containing the prepro-cecropinB2 gene sequence was provided by Professor Guangwu Du at National Chung Hsing University. The *E. coli* host strains and vectors used in this study are listed in Table 4. All other chemicals were analytical grade and obtained from a local dealer.

### 4.2. Construction of Expression Systems

**Figure 7:**
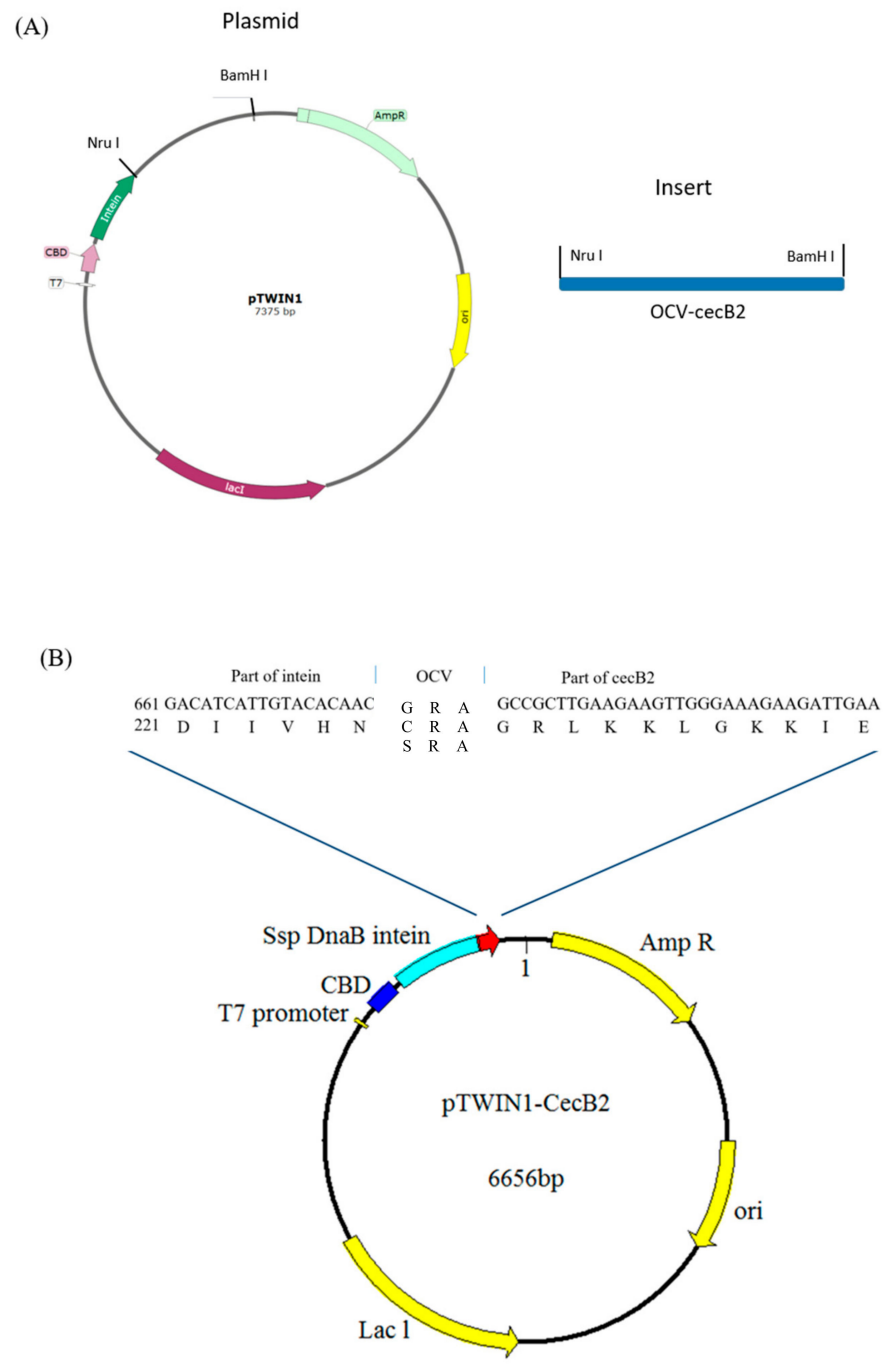
The plasmid and insert used in this study include (**A**) pTWIN1 and insert and (**B**) the construct of CBD-Int-OCV-cecbB2, where the OCVs were GRA, CRA, SRA, and the OCV amino acid number began from 227.

The prepro-cecB2 template was applied in PCR to amplify the cecB2 gene carrying various OCV sequences. The primers are listed in Table 1. The obtained inserts (PCR products) were further ligated to vector pTWIN1 (New England Biolabs, Ipswich, MA, USA) via the restriction sites NruI and BamHI to build the constructs of CBD-INT-OCV-cecB2, as shown in Figure 7, where the gene and amino acid sequences were numbered from the beginning codon of the CBD. All recombinant DNA manipulations were performed following standard procedures [33].

### 4.3. Cultivation Conditions

*E. coli* ER2566 hosts carrying various fusion protein genes were activated from preservation in a petri dish with 10 mL of LB culture agar medium plus 10 μL (50 mg/mL) ampicillin. Two colonies from the dish were picked to inoculate 5 mL LB medium plus 5 μL (50 mg/mL) ampicillin, followed by overnight cultivation at 37 °C and a shaking speed of 200 rpm. One milliliter of culture broth was used to inoculate 100 mL of LB culture medium plus 100 μL (50 mg/mL) ampicillin. Cultivation was conducted at 37 °C and 200 rpm until cell optical density (OD_600_) reached 0.6. Isopropyl β-D-1-thiogalactopyranoside (IPTG) was added to a concentration of 0.4 mM, and then cultivation continued at 15 °C and 200 rpm for another 16 h. The harvested cells were subjected to centrifugation and disruption for further protein analysis by sodium dodecyl sulfate-polyacrylamide gel electrophoresis (SDS-PAGE) and Western blot testing for CBD.

### 4.4. CecB2 Production

The harvested cell pellet was resuspended in Tris HCl buffer (20 mM Tris HCl, pH 8.0, 50 mM NaCl) and then lysed by sonication, using an ultrasonic processor at 20 W for 10 cycles (30 s working, 30 s free), to obtain the crude cell extract. The supernatant was collected and applied to a chitin column, followed by rinsing with the washing buffer (20 mM Tris HCl, pH 8.0, 100 mM NaCl, 0.1 mM EDTA, and 0.1% Triton X-100, Merck, Taiwan). The cleavage buffer (20 mM Tris HCl, pH 6.0, 50 mM NaCl, and 0.1 mM EDTA) was then loaded and the sample kept at 25°C overnight to obtain cecB2. The column was cleaned by loading stripping buffer (0.3 M NaOH) for 30 min to remove the bound CBD-INT segment. The eluted cecB2 was stored at 4 °C until further use. For the INT cleavage test, the crude cell extract was concentrated via a filter (Amicon Ultra-15, Merck Ltd., Taiwan, 10 kDa). The upper retentate was collected and mixed with the cleavage buffer under various cleavage conditions.

### 4.5. Assays

Protein analysis was carried out using 15% SDS-PAGE and Coomassie Blue staining [34]. The Tricine-SDS-PAGE gel was prepared with 2.82 mL, 16.6% separation gel, 0.6 mL, 7.5% spacer gel, and 0.5 mL, 3% stacking gel. The other procedures followed that reported by Schagger [35]. For CBD western blot analysis, the protein samples run on the SDS-PAGE were transferred to PVDF (polyvinylidene fluoride) membrane. After being blocked and washed, the membrane was incubated overnight with the primary CBD antibody (1:10000), followed by 2-h incubation with an anti-goat tag secondary antibody and stained using the BCIP/NBT chromogenic system (Boster, Pleasanton, CA, USA) [36]. The Bradford protein assay was used to measure total protein content [37], and imageJ software (version 1.49s) was used to determine each band’s area and color intensity [38]. The color intensity was defined as the band’s area multiplied by its intensity. The color intensity of the cleaved cecB2 band was measured and correlated to the protein concentration via a BSA standard curve. In the cleavage and kinetic tests, the color intensity of the cleaved segment was measured to represent the relative level of the protein concentration.

Antimicrobial activity was modified from the agar diffusion test [39]. In brief, a volume of 150 μL of tested bacteria dilution solution (OD_600_ = 0.7) was added to 6 mL of sterilized LB broth containing 0.8% agar, followed by pouring it into a petri dish (9.0 cm) to form an agar with a 1 mm depth. To prepare the sample, the purified AMP sample was diluted following the serial two-fold dilutions. In the test, a volume of 20 μL of the diluted sample was added to the surface of the agar and the petri dish was incubated at 37 °C for 12 h. Antimicrobial activity was observed by the clear zone around the testing spot. The minimal serial dilution number was set where no clear zone was observed. Each assay was performed in triplicate and the average serial dilution number was calculated. The minimum inhibition concentration (MIC) was defined as the least-diluted concentration processing antimicrobial effect [40].

### 4.6. Protein Modeling and in Silico MD Simulation

Prior to the MD simulation, the structural model of CBD-INT-OCVs-cecB2 fusion protein had to be built [17]. Based on the Phyre2 server [41], three structures were applied as templates—the structure of the CBD of chitinase A1 from *Bacillus circulans* WL-12 (PDB ID: 1ED7), the crystal structure of DnaB INT from *Synechocystis* sp. PCC 6803 (PDB ID: 1MI8), and the solution structure of the GK cecropin-like peptide from *Aedes aegypti* (PDB ID: 2MMM). The initial template of cecB2 was modeled via the Phyre2 server [42]. The ZDOCK server [35] was applied to predict the relative position between domains. The whole structure of CBD-INT-OCV-CecB2 protein was modeled using MODELLER 9v13 [43], and the resulting structure is in shown in Figure S1. Its stereochemical qualities were validated with ProSA [44] and PROCHECK [45] with the result shown in Figure S2. These validation results revealed that our model structure of CBD-INT-CecB2 was reliable for further studies.

After the protein model was built, it was subjected to an MD simulation using GROMACS version 4.6.7 with OPLS-AA force field in the TIP3P explicit solvent model [46]. The protein model was immersed in an orthorhombic water box (80.41 × 80.41 × 80.41 Å with the box angles of 60°, 60°, and 90°). The net charge was neutralized by the addition of sodium and chloride ions (at 150 mM salt concentration). Long range electrostatics were handled using the particle mesh Ewald method. Steepest descent energy minimization was used to remove possible bad contacts from the initial structures until energy convergence reached 1000 kJ/(mol·nm). The systems were subjected to equilibration at 300 K and normal pressure constant (1 bar) for 100 ps under the conditions of position restraints for heavy atoms and LINCS (Linear Constraint Solver) constraints. The time step of the simulation was set to 2 fs, and the coordinates were saved for analysis every 100 ps. During the MD simulation, the potential energy of the system converged after 30 ns, indicating that the simulation system had equilibrated. By monitoring the root-mean-square deviation (RMSD) of Cα atoms, the overall fold also became stable after 30 ns of simulation, suggesting that the protein model built by homology modeling was reliable and would not become denatured. As Cα RMSD and the secondary structure content became stable after 300 ns, the protein folding was considered nearly complete and the simulation was stopped at 400 ns. The final GRA variant structure after the 400 ns MD simulation was used for the model generation of the SRA and CRA variants.

The local motions of the OCVs (GRA, CRA, and SRA) were studied using MD simulation. The setup included a force field, solvent model, and the same parameters as the aforementioned 400 ns MD simulation for GRA. Each protein variant was subjected to a 20 ns MD simulation. After a 10 ns equilibration phase, the last 10 ns trajectories were analyzed using GROMACS utilities. The root-mean-square fluctuation (RMSF) was calculated using the rmsf_states.py script in PyMOL (http://pldserver1.biochem.queensu.ca/~rlc/work/pymol/). The solvent accessibility of the protein variants at residue level was calculated using POPS [47]. A 20% cutoff threshold line was used to define the exposure status, i.e., buried or exposed [19]. All structure figures were prepared using PyMOL [48].

### 4.7. Kinetics Models

A first-order kinetic model was used to describe intein cleavage kinetics, as follows:

(1) $$\frac{dc}{dt}=k\;c$$

where *k* is the kinetic rate constant in unit (*d*^−1^) and c is the intein concentration. The kinetic constant of Equation (1) with an initial condition of *c* = *c*_0_ (*t_0_* = 0) is expressed as

(2) $$k=\frac{\ln c(t)-\ln c_{0}}{t-t_{0}}$$

The first-order reaction kinetics is based on the reaction mechanism that only one reactant is involved in the reaction rate-limiting step, which is quite proper to describe the intein cleavage reaction as shown in Scheme 1. The regression was performed using Origin software V9.0 (OriginLab, Northampton, MA, USA). The intein cleaved part concentration was represented by the color intensity (the total pixel resolution of the band) in SDS-PAGE, which could be correlated to relative protein concentration.

### 4.8. Statistical Analysis

To obtain the statistical results, all data were analyzed by Origin software, version 9.0. Experiments were performed in triplicate and expressed in mean ± SD; the significant difference between means was identified via analysis of variance (Origin software, version 9.0).

## Figures and Tables

**Table 1:** Primers used in this study.

Name	Primer	Sequence (5′-3′)	Restriction Enzyme
CBD-INT-GRA-CecB2	GRA-F’	GTC GCG AAT GAC ATC ATT GTA CAC AAC GGC CGG GCG GGC CGC TTG AAG AAG	Nru I
	R’-GRA	CGC GGA TCC TTA TCT TCC TAG TGC CTG	BamH I
CBD-INT-CRA-CecB2	CRA-F’	GTC GCG AAT GAC ATC ATT GTA CAC AAC TGC AGA GCC GGC CGC TTG AAG AAG	Nru I
	R’-CRA	CGC GGA TCC TTA TCT TCC TAG TGC CTG	BamH I
CBD-INT-SRA-CecB2	SRA-F’	GTC GCG AAT GAC ATC ATT GTA CAC AAC AGC AGA GCC GGC CGC TTG AAG AAG	Nru I
	R’-SRA	CGC GGA TCC TTA TCT TCC TAG TGC CTG	BamH I

**Table 2:** Comparison of the cleavage rate constants and cecB2 yields for INT with various OCVs.

Item	GRA	CRA	SRA
Rate constant *k* (*d*^−1^) ^a^	0.292 ± 0.013	0.240 ± 0.044	0.595 ± 0.020
R-square	0.996	0.934	0.998

^a^ The rate constant was estimated via linearly fitting the graph of color intensity (ln) vs. time as listed in Equation (2), where the slope represents the rate constant, displayed with mean ± SD in dimension (*d*^−1^).

**Table 3:** **Serial dilution number** of the purified cecB2 against various *E. coli* strains.

Strain	Serial Dilution Number ^a^
*E. coli* JM109	5.0
*E. coli* DH5α	4.0
*E. coli* BL21	4.3

^a^ Serial dilution number was in two-fold series with the average of three duplicate discs.

**Table 4:** *E. coli* strains used in this study.

Strain or Plasmid	Genotype and Relevant Characteristics	Source
DH5α	F^–^ endA1 glnV44 thi-1 recA1 relA1 gyrA96 deoR nupG Φ80d*lac*Z △M15 △ (*lac*ZYA-argF)U169, hsdR17(r_K_^−^ m_K_^+^) λ–	Novagen, USA
ER 2566	F-λ- fhuA2 [lon] ompT lacZ::T7 gene 1 gal sulA 11 △(mcrC-mrr)114::IS10R (mcr-73::miniTn10-TetS)2 R(zgb-210::Tn10)(TetS)endA1 [dcm]	New England Biolabs, USA
T-vector	pBluescript IISK(−) with modified MCS	Yeastern biotech
pTWIN1	Cloning vector. Amp	New England Biolabs, USA

## Supplementary Materials

The following are available online at https://www.mdpi.com/1420-3049/25/4/1005/s1, Figure S1: Predicted structure of CBD-INT-CecB2 fusion protein. Figure S2: The quality assessment of CBD-INT-CecB2 fusion protein. Table S1: Abbreviation table.
