# Supplementary material for: Study on Cecropin B2 Production via Construct Bearing Intein Oligopeptide Cleavage Variants
Source: Molecules. 2020 Feb 24;25(4):1005. doi: 10.3390/molecules25041005 (PMC7070832; doi:10.3390/molecules25041005)
Supplement: Supplementary file 1 [file molecules-25-01005-s001.pdf]

molecules

Supplementary Materials

Study on Cecropin B2 Production via Construct Bearing Intein Oligopeptide Cleavage Variants

Yi-Ting Fang [1], Si-Yu Li [1,2], Nien-Jen Hu [3], Jie Yang [3], Jyung-Hurng Liu [4,5,6,*] and Yung-Chuan Liu [1,*]

1 Department of Chemical Engineering, National Chung Hsing University, Taichung, 402, Taiwan; tanya19931126@gmail.com (Y.-T.F.); syli@dragon.nchu.edu.tw (S.-Y.L.)
2 Innovation and Development Center of Sustainable Agriculture, NCHU, Taichung, Taiwan
3 Graduate Institute of Biochemistry, National Chung Hsing University, Taiwan; njhu@nchu.edu.tw (N.-J.H.); jieyang1110@gmail.com (J.Y.)
4 Institute of Genomics and Bioinformatics, NCHU, Taichung, Taiwan
5 PhD program in Medical Biotechnology, NCHU, Taichung, Taiwan
6 Department of Life Sciences, NCHU, Taichung, Taiwan
* Correspondence: jhliu@dragon.nchu.edu.tw (J.-H.L.); ycliu@dragon.nchu.edu.tw (Y.-C.L.); Tel.: +886-4-22853769 (Y.-C.L.); Fax: +886-4-22854734 (Y.-C.L.)

1. Amino acid sequence for cecropin B1:

SRAGRLKKLGKKIERAGKRVVNAAQKGLPVAAGIQALGR

2. Gene sequence for cecropin B1:

AGCAGAGCCGGCCGCTTGAAGAAGTTGGGAAAGAAGATTGAACGGGCGGGCAAGCG TGTCGTTAATGCCGCTCAGAAGGGACTCCCCGTTGCAGCTGGCATCCAGGCACTAGGAAGA

3. Abbreviation Table

Table 1. Abbreviation table.

Abbreviation	Definition
AMP	antimicrobial peptide
cecB2	cecropinB2
CBD	chitin binding domain
INT	Ssp DnaB intein
IPTG	Isopropyl β-D-1-thiogalactopyranoside
MD	molecular dynamics
MIC	minimum inhibition concentration
OCVs	oligopeptide cleavage variants
OD600	optical density at 600nm
RMSD	root-mean-square deviation
RMSF	root mean square fluctuation

4. Supplementary Graphs:

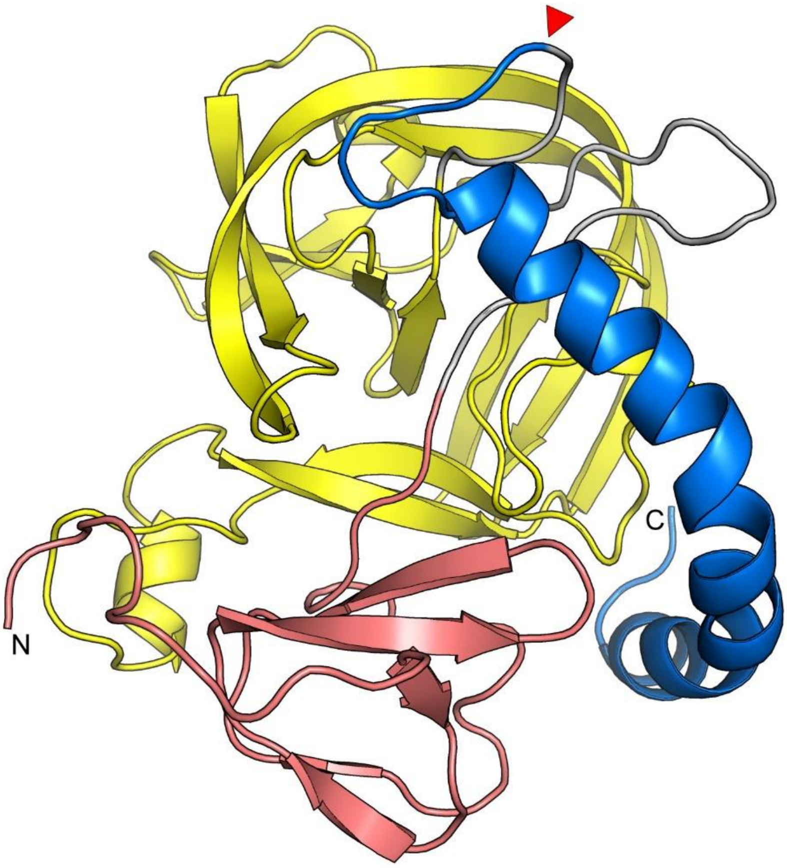

Figure 1. Predicted structure of CBD-INT-CecB2 fusion protein. The fusion protein is shown in ribbon presentation. The domains (CBD, INT, and CecB2) are colored as pink, yellow, and blue, respectively. The linker sequences are colored in grey. The location of intein cleavage site is marked as a red triangle.

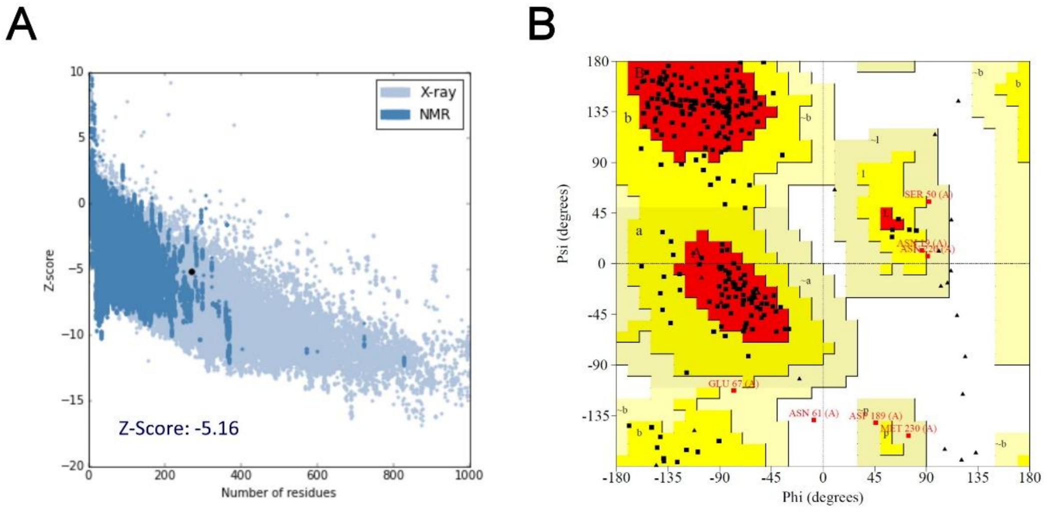

Figure S2. The quality assessment of CBD-INT-CecB2 fusion protein. (***A***) Z-score plot generated by ProSA. The z-score value of the final model structure is -5.16 and the plot indicates that the overall model quality is within the range of scores typically found for proteins of similar size. (***B***) Ramachandran plot obtained by PROCHECK. Most favored regions: 78.5% of residues in the final structure; additional allowed regions: 18.6%; generously allowed regions: 2.5%; disallowed regions: 0.4%. This protein model has an overall G-factor of −0.88. This score is above the lower limit (–1.0) considered as non-acceptable, indicating that the backbone architecture of this model is acceptable.
